# Thank You to Our 2022 Peer Reviewers

**DOI:** 10.1029/2023GH000857

**Published:** 2023-05-21

**Authors:** Gabriel Filippelli, Rita R. Colwell, Susan Anenberg, Daniela Ceccarelli, Meredith Franklin, Sagnik Dey, Karen A. Hudson‐Edwards, Antarpreet Jutla, Chiyuan Miao, Adina Paytan, Avner Vengosh

**Affiliations:** ^1^ Indiana University—Purdue University Indianapolis Indianapolis IN USA; ^2^ University of Maryland College Park MD USA; ^3^ George Washington University Washington DC USA; ^4^ Research Executive Agency (REA) Bruxelles Belgium; ^5^ USC Los Angeles Los Angeles CA USA; ^6^ Indian Institute of Technology Delhi New Delhi Delhi India; ^7^ Camborne School of MInes Environment and Sustainability Institute University of Exeter Penryn UK; ^8^ University of Florida Gainesville FL USA; ^9^ Beijing Normal University Beijing China; ^10^ UCSC Santa Cruz CA USA; ^11^ Division of Earth and Climate Sciences Levine Science Research Center Duke University Durham NC USA

## Abstract

- The editors thank the 2022 peer reviewers
- In 2022, GeoHealth benefited from 333 reviews provided by 245 of our peers
- A number of individuals submitted multiple reviews for GeoHealth in 2022

Peer review is at the heart of the scientific endeavor, ensuring that high‐quality discoveries are communicated in effective and impactful ways. As a voluntary and mostly anonymous effort, peer review is often poorly recognized. But it is so valuable to journal Editors, and we are often so impressed by the incredibly detailed, constructive, and informative reviews that we get back from reviewers. In 2022, GeoHealth benefited from 333 reviews provided by 245 of our peers for papers submitted to the journal. Thank you all for being such an important part of the scientific process, advancing the communication of discoveries at the intersections of the environmental and health sciences to improve society.

Thank you to the 245 reviewers who submitted 333 reviews in the journal last year. Individuals in italics provided two or more reviews for GeoHealth during the year.

*Abadi, Azar*

Adeyemi, Olusola

Agyekum, T

*Ahn, Yoonjung*

Akanda, Ali Shafqat

Alahmad, Barrak

Ardon‐Dryer, Karin

Argyraki, Ariadne

Asfaw, Zamede

Awasthi, Shubham

*Bai, Zhaohai*

Bandaragoda, Christina

*Barkjohn, Karoline*

Barnard, Malcolm A.

*Barros, Marcia*

Benmarhnia, Tarik

Berman, Jesse

*Bernhardt, Jase*

Bernstein, Aaron

*Black, Alan*

*Bostick, Benjamin C.*

*Bröde, Peter*

Bratburd, Jenny

Breil, Margaretha

Buonocore, Jonathan J.

*Cai, Wenjia*

*Castillo, Maria D.*

Censi, Paolo

Chakraborty, Jayajit

Chambliss, Sarah

Charlet, Laurent

*Chen, Kai*

Chen, Kaiyu

Ching, Joseph

Chowdhury, Sourangsu

Clark, Pat

Conibear, Luke

Conley, Jamison

Coyte, Rachel M.

*Cruz, Miguel*

Cui, Qianqian

Cutter, Susan L.

Davidson, Anna

*DeSchrijver, Evan*

*Dhiman, Ramesh C.*

Di Napoli, Claudia

Du, Tien L.

Duke, Clifford

Entwistle, Jane A.

Fan, Xiaolong

Fang, Liancheng

*Ferrara, Vincenza*

*Ferreira, Francisco*

Field, William

Fiffer, Melissa

*Fomba, Khanneh Wadinga*

Fortner, Sarah

*Gao, Yang*

Garcia‐Menendez, Fernando

Garrido Cumbrera, Marco

Gassó, Santiago

Glaeser, Stefanie

*Gohlke, Julia M.*

González‐Marín, Alicia

*Gorla, David*

Gueye, Moussa

*Guimarães, Ricardo J.*

Gupta, Lovleen

*Gutschick, Vincent P.*

Han, Ling

*Hand, Jenny L.*

*Harp, Ryan D.*

Hart, Robbie

He, Mike

Heck, Julia

Heft‐Neal, Sam

Heindel, Ruth C.

Hendrix, Donald A.

Hendryx, Michael

*Herckes, Pierre*

Hon, Kai Kwong

Hovel, Rachel A.

*Hsieh, Shizuka*

Huang, Guanghui

Huang, Jianping

Hudson, Paul

*Jagai, Jyotsna*

Jalalzadeh Fard, Babak

*Jamro, Imtiaz Ali*

*Johnson, Daniel*

Johnston, Nancy A.

Jordan, Kurt

*Joseph, Naveen*

Karl, David M.

Kavouras, Ilias

Kaynak, Burcak

*Ke, Ziming*

Kelly, Jamie

*Kelp, Makoto*

*Kerekes, John*

*Kjaer, Lene Jung*

Kleiman, Gary

Knappett, Peter S.

Kong, Dongxian

Krauchanka, Julia

La Mantia, Tommaso

Laidlaw, Mark

Lapen, David R.

*Lee, Michael J.*

Li, Guoxing

*Li, Longxiang*

Li, Meifang

Liang, Fengchao

Liao, Chuan

Liao, Weilin

Lim, Chris

Liu, Dan

Liu, Qi

Liu, Xue

Liu, Yisi

*Liu, Yongwen*

Lopes, Hélder D.

*Love, Mark W.*

Ma, Yiqun

Macaulay, Elizabeth

*Machete, Ines*

*Maier, Andreas*

Malings, Carl

Malley, Chris

Marais, Eloise A.

*Marquis, Benjamin*

Mbandi, Andriannah

McDuffie, Erin

McMillan, Joseph

Moreno Madrinan, Max J.

Morgan, Geoffrey

Morrissey, Margaret C.

*Nachman, Keeve E.*

Nath, Debashis

Ncipha, Xolile

Newman, Peter

*Nicely, Julie M.*

*Nishant, Nidhi*

Norton, David

Nui, Li

*O'Dell, Katelyn*

*Ogunjo, Samuel T.*

Orimoloye, Israel R.

*Ostro, Bart*

*Palinkas, Lawrence A.*

Paul, Chris

*Phuong, Jimmy*

Pleim, Jonathan E.

Pope, Francis D.

*Pope, Richard J.*

Pope III, C. Arden

Porcasi, Ximena

*Ramirez, Ivan J.*

Ramirez‐Andreotta, Monica

*Ransom, Katherine M.*

Rao, Neethi

Rastogi, Deeksha

*Raymond, Colin*

Raysoni, Amit U.

Reid, Andrea

Rizzo, Luciana V.

*Rogan, Eleanor*

*Ru, Muye*

Saari, Rebecca

*Sagnotti, Leonardo*

Sahagian, Dork L.

Salako, Kazeem A.

Salyer, Stephanie

Sandifer, Paul A.

*Sant'Ovaia, Helena*

Santos, Jefferson P.

Sarofim, Marcus

Scheckel, Kirk

*Schreiber, Madeline*

Scibior, Agnieszka

Scudero, Salvatore

*Sealy, Andrea*

Semmens, Kathryn

*Sera, Francesco*

Shi, Liuhua

*Shi, Zitong*

Simeonov, Vasil

Skelly, Sonja

Smith, Allan H

Smith, Kelly H.

Smith, Ted

Snorek, Julie

*Soleri, Daniela*

*Spear, Robert*

*Stowell, Jennifer D.*

Sun, Peng

Sun, Qingqing

*Tahir, Muhammad*

Tee Lewis, P. Grace

Teixidor‐Toneu, Irene

Thakrar, Sumil

*Thilakaratne, Ruwan*

*Thompson, Segayle*

*Tong, Daniel*

TS, Anish

Turner, Alexander J.

Turner, Nancy J.

Ueda, Kayo

Uejio, Christopher

*Usmani, Moiz*

Utembe, Wells

Varotsos, Konstantinos V.

*Vecellio, Daniel J.*

Vieira Barbosa, Joana

Viveiros, Fátima

Vohra, Karn

Wadinga Fomba, Khanneh

*Wang, Dongchuan*

*Wang, Sally*

Wang, Suwei

Wangdi, Kinley

Weber, Rodney J.

Wells, Ellen

Willenbring, Jane

Wilmer, Hailey

*Wimberly, Michael C.*

*Wowk, Kateryna*

*Wu, Junya*

*Wu, Mingxuan*

*Wu, Shiliang*

*Wu, Wei*

Xi, Yuzhi

*Yan, Xing*

Yang, Yuanjian

You, Yongfa

Yue, Songshan

*Zaitchik, Benjamin F.*

Zhang, Charlie

Zhang, Jianjun

Zhang, Peng

Zhang, Qingli

Zhang, Wenjie

Zheng, Sheng

*Zhu, Kevin*

Zuddas, Pierpaolo

